# Fecal Impaction

**Published:** 1905-01

**Authors:** C. M. Rickert

**Affiliations:** Harrisburg, Pa.


### FECAL IMPACTION.

BY C. M. RICKERT, M.D., HARRISBURG, PA.

With a full knowledge of the anatomy and physiology of the alimentary canal, and the many deviations from the normal due to some pathological change with adhesions, we are surprised at the comparatively few cases of obstruction of the bowels, due to impaction of the feces, which occur. The ingesta meets with resistance at all of the flexures and hence we find impactions more likely at the cecum, sigmoid and rectum. On account of the greater resistance at the flexures, we find them the seat of disease more frequently than anywhere else—and the sigmoid possibly the most frequent. The rectum is nothing more than a receptacle for the feces and the sigmoid flexures acts as a support of the fecal mass in the lower colon, hence it is plainly evident why impaction is more liable at this point than anywhere else. In constipation, due to some local trouble like a painful fissure or perhaps some other cause, we are occasionally called to treat an impaction in the rectal pouch. We find at times a very hard mass, stone-like, no doubt due to the rapid absorption of the fluids and the constant pressure from above.

*The Causes* of impaction are numerous, principally catarrhal diseases and dilatation of the colon, foreign bodies, as tomato or grape seeds, fruit pits, enteroliths and worms. In children the cause is generally worms.

*The Symptoms* are not always plainly recognizable at first. There may be few subjective symptoms for a considerable time. The patient may have constipation followed by colicky pains and diarrhea, the stools generally containing mucus and blood. We may then find an enlargement which sometimes is readily defined, as it may be of doughy consistence and have somewhat the shape of the bowels. There is autointoxication with its train of symptoms, viz., coated tongue, fetor of breath, fever, vomiting and sometimes great mental disturbances. There may be pain in the region of the impaction due to the irritation and inflammation of the bowel.

*The Diagnosis* is not always plain. When there is impaction in the rectum, where an examination is comparatively easy, there is not so much difficulty. In persons with a thin abdominal wall, it is sometimes possible to detect the shape and consistency of the mass. But where the tumor is not easily recognized and on account of the color of the patient, due to infection, it may easily be mistaken for some malignant disease; indeed, I have found both. In two of my cases lately, malignant disease wàs suspected, a number of the symptoms being alike in both. In the one case it soon became evident that our suspicions were well founded, and in the other we dismissed the suspicions just as quickly, for the patient improved at once.

*Illustrative Cases.*—I cite the history of only two of my cases to which I was called in consultation lately:

No. 1—Male, fifty-two years old. Was in fair health for years; within the last two years he suffered from attack of malaise and slight fever, diagnosed as malarial. During the last year these attacks increased in frequency and severity, accompanied by pain in the region of the umbilicus. Later noticed a hardness in abdominal wall extending from the sigmoid to the umbilicus. Constipation had existed for some years, but patient had used salines daily and had small watery stool. Had an anal fissure treated by local applications about eight years ago. When I was called in consultation patient had been in bed for three weeks suffering a great deal of pain, swelling in abdomen quite perceptible and tender to touch, temperature 103°, furred tongue, very nervous and other symptoms of a general infection. I found the fissure still present, having been a source of constant irritation so that the sphincters were spasmodically closed, that a finger could be introduced with the greatest difficulty. The patient was anesthetized, and after some trouble the hypertrophied sphincter muscle thoroughly divulsed, so that the condition at the sigmoid could be carefully examined, for I suspected fecal impaction. With the sigmoidoscope I discovered a hardened mass of fecal matter, blood and mucus. I broke up the part that could be reached and after four flushings at different times, I removed about three quarts of fecal matter, some very hard and gritty and the other semi-solid. The odor was something never to be forgotten and as there was an open surface at the fissure due to the stretching and division of the superficial muscle fibers, I feared infection by these discharges and was very careful to protect the parts with iodoform and kept them thoroughly aseptic. Several superficial abscesses formed, however, and recovery was slow, but complete. You will note in this case there were daily evacuations, by the use of salines, notwithstanding the fact that the sigmoid and descending colon were filled with this large fecal mass.

No. 2.—Male, fifty-six years old; enjoyed good health until about five months ago. He commenced to have trouble with constipation, followed by diarrhea. Had quite a great deal of flatulence, pain in the region of the sigmoid and occasional fullness. Then attacks of vomiting accompanied by severe colicky pains.

At the time I was called in, he had suffered from such an attack for two days with no movement of the bowels; medicines were given by the mouth to move bowels and enemas were tried, but of no avail. The abdomen was highly tympanitic, vomiting was becoming suspicious. I examined patient and decided there was obstruction at the sigmoid flexure. The rectum had been emptied by the numerous enemas, so I tried to introduce a bougie. After about an hour's hard work, I was successful in introducing a No. 5, Wales bougie, and injected about two quarts of hot borax solution. After patiently waiting for about six hours, the bowels started to move and within the next hour the patient had about twenty stools of tomato seeds and semi-solid fecal matter. The tomato seeds had been arrested and formed a solid mass, the wife of the patient affirming that about a pint of seed were passed. There was thickening of bowel with a narrowing of the lumen, with a few other symptoms. I suspected malignant trouble.—*The Medical Council.*

